# Translational Challenges in Cancer Nanotherapy

**DOI:** 10.34172/apb.2024.021

**Published:** 2023-10-25

**Authors:** Ammu V. V. V. Ravi Kiran, Garikapati Kusuma Kumari, Praveen T. Krishnamurthy, Bhadram Kalyan Chekreverthy

**Affiliations:** ^1^Department of Pharmacology, JSS College of Pharmacy (JSS Academy of Higher Education & Research), Ooty, The Nilgiris, Tamil Nadu 643 001, India.; ^2^Department of Pharmaceutical Analysis, JSS College of Pharmacy (JSS Academy of Higher Education & Research), Ooty, The Nilgiris, Tamil Nadu 643 001, India.

## To Editor,

 For decades, cancer has been a long-standing disease, globally. The current treatment options including chemotherapy, radiotherapy, etc. are failing due to tumor resistance, relapse and treatment-associated off-target side effects.^1^ Moreover, the tumor fort i.e., tumor microenvironment (TME) has emerged as a barrier, aiding the tumors in achieving their hallmarks.^2^ In recent years, nanotechnology has paved the way for solving many problems majorly treatment-associated off-target side effects, which has gained the attention of many biomedical engineers and scientists.^2,3^ Despite having many advantages, nanotechnology has also posed serious toxicological problems, especially in clinical trials, creating hesitation among industrial collaborations.

 Currently approved cancer nanotherapeutics (such as Abraxane^®^) suffer from many complications including non-specificity, and obstruction by tumor fort, resulting in poor efficacy.^2^ Incorporating certain strategies in nanomedicine would favour the shift from bench-side to bedside.

## Challenges of cancer nanomedicine

 Having many advantages, nanoparticles are limited by toxicity-related issues, ROS generation, protein corona effect, etc. The following are the important challenges in nanomedicine that need to be addressed.

## Biological challenges

###  Protein/biomolecules corona effect – impact on pharmacokinetic and pharmacodynamic

 Upon entry into the physiological system, nanoparticles interact with many biological components and are coated with proteins, lipids or carbohydrates, impacting their distribution and efficacy. Protein/biomolecule coronas influence cellular uptake and internalization, especially in targeted therapies. Biological free proteins hide the targeting moiety thereby rendering them similar to passive targeting. Further, these interactions could also alter the particle size and surface charge of nanoparticles. Improper particle size could interfere and disrupt many biological membranes, and protein folding.^2,4,5^ These disruptions could generate many immunogenic reactions (e.g., frustrated phagocytosis), especially by immune cells like macrophages or monocytes. Similarly, positively charged nanoparticles can influence the surface dynamics and result in disruption causing cell death. For example, ultrasmall gold nanoparticles (i.e., size 1-4 nm) could block voltage-gated potassium channels, resulting in cardiac problems.

 The efficiency of gene transfection has greatly improved especially with the utilization of suitable polymers (esp. with positively charged polymers). Positively surface charge not only enables the attachment of genetic agents but also helps in escaping lysosomal degradation via the proton sponge effect. Surface charge variation in nanoparticles could result in toxic effects such as disruption of cells from the site of entry to the target region.^2,4,5^

###  Tumor microenvironment (TME): The tumor fort

 TME is one of the important obstacles for nanoparticles to bypass. TME acts as a fort surrounding tumors; and comprises both cellular and non-cellular components that influence the entry and efficacy of nanoparticles.^2^ These corrosive TME and lysosomal degradations could disintegrate nanoparticles, which could result in the generation of oxidative stress. Selection of suitable nanoparticles is important since the nature of nanoparticles could promote toxicities when reacted with tumor fort. Most metals and carbon nanomaterials could bypass the acidic TME but promote ROS which could promote tumor metastasis.^2^

 Stimuli-sensitive nanoparticles have been created to overcome the tumor fort. Although successful in the preclinical setting, these nanoparticles are expected to influence normal physiology due to the similarity of TME components.^2^ For example, pH-sensitive nanoparticles are ineffective in gastric or vaginal cancers due to their physiological pH <4. The microenvironment in the aged population differs from the young population. The young TME comprises robust immune cells, and fibroblasts, which are inadequate in aged TME. In the preclinical setting, the aged microenvironment is much ignored, as most testing was carried out in young rodent models.^2^

###  Immunogenicity 

 Nanoparticle surfaces modified with polymers such as PEG reduce protein corona and prolong the circulation times. Studies have shown that coating PEG would result in hypersensitivity reactions and anaphylaxis due to the generation of anti-PEG IgG antibodies. Additionally, these anti-PEG IgG antibodies could activate the complement activation and complement-independent pseudo-allergies.^5^

## Manufacturing and testing challenges

 Achieving controllable and reproducible synthesis of nanoparticles is crucial for successful clinical translation. Nevertheless, the quality assessments of each property of nanoparticles remain difficult, especially in batch-to-batch scenarios. Upon rapid development of nanoparticles, evaluation of biocompatibility at the cellular level is essential before testing at the preclinical level.^6^ However, *in vitro* models are limited by the fluid flow and complexity of tissues. Utilization of ‘organ/tissue/tumor-on-a-chip’ tools has helped in overcoming the limitations of *in vitro* models and helped in the proper correlation of nanoparticles with *in vivo* models.^7^ Further, the performance of nanoparticles in *in vivo* models is obligatory, especially for assessing the pharmaco-kinetic and -dynamics. Replication of human tumors in animals is still in infancy due to their inability to reproduce all aspects of cancers including heterogeneity, EPR effect, and metastasis.^6^

 Upon successfully developing and optimizing the nanoformulation, clinical translation has a major challenge in scaling up the product. Scaling manufacturing has complex processes i.e., chemical synthesis, manufacturing, controlling (CMC) along with good manufacturing practices (GMP), and commercialization.^6^ In comparison with simple nanoparticles, complex nanomedicines pose extensive and additional GMP challenges and require continuously updated knowledge.

## Outlook

 Overall, nanomedicine has proved to be effective in treating many incurable cancers, especially in preclinical settings. The translation of nanoparticles from bench-side to bedside is not straightforward due to their intricate designs and complex CMC and GMP systems. From a regulatory point of view, stringent checks have to be performed especially in monitoring the physiochemical properties, stability, batch-to-batch variation, pharmacokinetic and pharmacodynamic, and toxicology of nanoparticles. Addressing these challenges would greatly reshape the cancer treatment options and further facilitate clinical translation. Nevertheless, proper industrialization of cancer nanomedicines could only be possible by gaining an in-depth understanding of tumors as well as nanoparticles.

## Competing Interests

 None declared.

## Ethical Approval

 Not applicable.

## References

[1] Shi J, Kantoff PW, Wooster R, Farokhzad OC (2017). Cancer nanomedicine: progress, challenges and opportunities. Nat Rev Cancer.

[2] Ravi Kiran A, Kusuma Kumari G, Krishnamurthy PT, Khaydarov RR (2021). Tumor microenvironment and nanotherapeutics: intruding the tumor fort. Biomater Sci.

[3] Wagner V, Dullaart A, Bock AK, Zweck A (2006). The emerging nanomedicine landscape. Nat Biotechnol.

[4] Oh JY, Kim HS, Palanikumar L, Go EM, Jana B, Park SA (2018). Cloaking nanoparticles with protein corona shield for targeted drug delivery. Nat Commun.

[5] Yang W, Wang L, Mettenbrink EM, DeAngelis PL, Wilhelm S (2021). Nanoparticle toxicology. Annu Rev PharmacolToxicol.

[6] Metselaar JM, Lammers T (2020). Challenges in nanomedicine clinical translation. Drug DelivTransl Res.

[7] Tian C, Zheng S, Liu X, Kamei KI (2022). Tumor-on-a-chip model for advancement of anti-cancer nano drug delivery system. J Nanobiotechnology.

